# Predicting High-Density Polyethylene Melt Rheology Using a Multimode Tube Model Derived Using Non-Equilibrium Thermodynamics

**DOI:** 10.3390/polym15153322

**Published:** 2023-08-07

**Authors:** Pavlina C. Konstantinou, Pavlos S. Stephanou

**Affiliations:** Department of Chemical Engineering, Cyprus University of Technology, P.O. Box 50329, 3603 Limassol, Cyprus; pac.konstantinou@edu.cut.ac.cy

**Keywords:** rheological model, polymer melts, non-equilibrium thermodynamics, multiple modes, normal stress coefficients, high-density polyethylene

## Abstract

Based on the Generalized bracket, or Beris–Edwards, formalism of non-equilibrium thermodynamics, we recently proposed a new differential constitutive model for the rheological study of entangled polymer melts and solutions. It amended the shortcomings of a previous model that was too strict regarding the values of the convective constraint release parameter for the model not to violate the second law of thermodynamics, and it has been shown capable of predicting a transient stress undershoot (following the overshoot) at high shear rates. In this study, we wish to further examine this model’s capability to predict the rheological response of industrial polymer systems by extending it to its multiple-mode version. The comparison with industrial rheological data (High-Density Polyethylene resins), which was based on comparison with experimental data available in (a) Small Amplitude Oscillatory shear, (b) start-up shear, and (c) start-up uniaxial elongation, was noted to be good.

## 1. Introduction

As reported by the Society of Plastics Industries (SPI) in 2000, the plastic industry in the US is positioned, in terms of shipment, in fourth place among manufacturing industries, following motor vehicles and equipment, electronic components and accessories, and petroleum refining [1]. A more recent survey predicts that the global plastic packaging market will be worth $269.6 billion by 2025, achieving a compound annual growth rate (CAGR) [2]. This figure alone highlights the impact of plastic materials on our lives and, thus, the significance of optimizing polymer processing technology. Future polymer processing will focus not on the machine, but on the product [1]. Several instabilities appear in the polymer industry, which makes life difficult for polymer engineers. For example, under certain circumstances, when molten plastic is forced through a die, the shark-skin defect appears [3]. To avoid this issue, it is suggested to slow down the manufacturing rate; however, this action decreases the production rates of commercial products, leading to an increase in cost. Wang et al. have suggested that this defect may be related to a molecular instability that corresponds to an oscillation of the absorbed chains in the die exit area between coiled and stretched states [4]. Thus, it seems that the answer needed should be sought by maintaining the molecular level of description and performing molecular dynamics simulations. The ultimate goal of this process is to predict the properties of a product via numerical simulations based on molecular principles and multiple-scale techniques [1].

Due to computational limitations, however, this goal has been unachievable until the last few years, in which period the extended evolution of simulation algorithms, the parallelization of these algorithms and the race, which has been very recently undertaken, to construct accurate coarse-grained potentials (directly derived from the atomistic simulations) have revolutionized the field. For example, by topologically and dynamically mapping atomistic simulation results onto the tube notion of the de Gennes–Edwards model, we have recently been able [5,6] to obtain the most fundamental function of the tube (reptation) theory (according to which the polymer motion due to entanglements is confined within a tube-like region, the axis of which coincides with the primitive path of the chain, and its diameter provides a measure of the strength of the topological interactions), namely the segment survival probability function, compare the atomistic simulations results against the predictions of modern tube models [7]; and even propose modifications to improve these models on a molecular level [8,9].

Accurate continuum simulations (usually using a finite element scheme [10]) require the use of accurate constitutive models, which are able to provide the necessary molecular physics associated with the rheological behavior of polymeric systems. Thus, the use of empirical models or models without reference to molecular physics may fail to represent even the qualitative features of the material’s behavior. Furthermore, a rather small set of parameters should be included in said models, to which a physical significance must be assigned, and the models should have the capacity to simultaneously fit all given data using a single set of parameter values [11]. Only in this context would polymer engineers be able to correctly predict the rheological response in industrial processes and solve several long-standing problems that the industry faces.

However, polymers exhibit a wide spectrum of relaxation times, which gives polymeric fluids a partial memory [12]. Conformation tensor-based models that include only a single mode cannot describe small-amplitude oscillatory shear (SAOS), in which a spectrum of relaxation times is needed. Even for dilute solutions, both theory and experiments suggest that a superposition of several exponential modes is obtained [13]. In the past two decades or so, several researchers employed multiple modes of well-known models in order to improve their predictive capabilities. For example, the Kaye-Bernstein–Kearsley–Zapas (K-BKZ) integral model [14,15], the Phan-Thien and Tanner (PTT) model [15,16,17,18], and the Giesekus model [15,18] have been used to predict the rheological behaviors of industrial polymers, such as low-density polyethylene (LDPE) and high-density polyethylene (HDPE). Although such well-known rheological models are able to reproduce experimentally observable features of the material functions in various flows, they fail to capture the correct physics.

Polymers with large molecular weights should be described based on the use of the tube theory mentioned above, which introduces terms such as reptation, chain contour length fluctuations, and constraint release (CR) due to the motion of surrounding chains [19]. Under flow, as polymer chains are oriented, a number of entanglements are expected, on average, to be lost, as dictated by the convective constraint release (CCR) mechanism [20,21] and shown to be the case via detailed atomistic non-equilibrium molecular dynamics (NEMD) simulations [22]. More recently, tube models [23,24,25,26] have been used to predict the appearance of a transient stress undershoot (following the overshoot) at high shear rates in start-up shear, which originated from the molecular tumbling of polymer chains in simple shear. Tube models have also been generalized to account for branches, such as the pom-pom model [27], as well as its thermodynamically admissible version, known as the pom-pon [28] model. Also, several works employed multimode versions of well-known tube models to predict the rheological responses of industrial polymer systems (we only mention a few of these works). Inkson et al. [29] used a multimode version of the pom-pom model and found that it can quantitatively address the rheology of LDPE for shear, uniaxial, and planar elongation. Soulages et al. [30] investigated the lubricated flow of a LDPE in a cross-slot geometry and compared the predictions of the extended pom-pom model [31] and the modified extended pom-pom model [16] to a plethora of rheological data: in shear, they compared it to transient and steady-state shear viscosity and the first normal stress coefficient, as well as the steady-state second normal stress difference, and in uniaxial elongation, they compared it to the transient uniaxial elongational viscosity. They noted that both models performed equally well (note that the thermodynamic admissibility of these two models is shown in Ref. [32]). Hoyle et al. [33] evaluated the performance of the multimode pom-pom model to those of both LDPE and branched HDPE melts, whereas, more recently, Konaganti et al. [15] employed the double convected pom-pom [34] model to predict the rheological behavior of a high-molecular-weight HDPE melt. Since multimode versions have been found to be superior to single-mode versions, our aim in this paper is to generalize the tube model of Stephanou et al. [23] to its multimode version and use it to predict the rheological response of a HDPE melt. 

The structure of the paper is as follows: In Section 2, the new model is briefly derived using non-equilibrium thermodynamics (NET). In Section 3, we derive the expressions of the relevant rheological material functions obtained by analyzing the asymptotic behavior of the model in the limits of small shear rates. The results obtained with the new model are then presented in Section 4, in which we first discuss its parameterization and then show how accurately and reliably it can describe the viscoelasticity of HDPE polymer melts. The paper concludes with Section 5, in which the most important aspects of our work are summarized, and future plans are highlighted and discussed. 

## 2. The Constitutive Model

### 2.1. State Variables

This work considers an isothermal and incompressible flow, meaning that the total mass density *ρ* and the entropy density (or temperature) are excluded from the vector of state variables. To characterize the polymer chains, the entanglement strand conformation tensor **c**, following the method of Stephanou et al. [23,35], is considered to be made dimensionless through $\tilde{c}=Kc/k_{B}T$, where *K* denotes the spring constant of the Hookean dumbbells that represents the entanglement strands at equilibrium, *k_B_* the Boltzmann constant, and *T* denotes the absolute temperature [36]. The conformation tensor $\tilde{c}$ refers to one entanglement strand, and at equilibrium (zero flow field applied), it coincides with the unit tensor. To characterize the multiple modes of the polymer chains, *N* conformation tensors are considered, with one tensor being considered for each mode [36]. Finally, the momentum density **M**, which is the hydrodynamic variable, is further considered, meaning that, overall, the vector of state variables is expressed as $x=\{M,c^{\left(1\right)},c^{\left(2\right)},\ldots ,c^{\left(i\right)},\ldots ,c^{\left(N\right)}\}$.

### 2.2. System Hamiltonian 

The mechanical part of the system’s Hamiltonian is given as [36]

(1) $$H_{m}\left(x\right)=K_{en}\left(x\right)+A\left(x\right)$$

where

(2) $$K_{en}(x)=\int \frac{M^{2}}{2\rho }dV$$

represents the kinetic energy of the system, whereas [23,35,36]

(3) $$A(x)=\int a(x)dV=\frac{1}{2}\sum_{i=1}^{N}\int G_{e}^{\left(i\right)}\left[\Phi \left(\mathrm{tr}\left({\tilde{c}}^{\left(i\right)}-I\right)\right)-\ln\det{\tilde{c}}^{\left(i\right)}\right]dV$$

represents the system’s Helmholtz free energy (with a(x) the Helmholtz free energy density) that includes the following contributions: the dimensionless potential $\Phi \left(\mathrm{tr}\left({\tilde{c}}^{\left(i\right)}-I\right)\right)$, which accounts for chain stretching, and an entropic contribution, which involves the logarithm of the determinant of the conformation tensor of each mode [35,36]. Here, $G_{e}^{\left(i\right)}$ is the entanglement modulus of the i^th^ mode, and **I** is the unit tensor. The partial derivative of the potential with respect to the trace of the conformation tensor defines the (dimensionless) effective spring constant [37,38] for the i^th^ mode

(4a) $$h\left(\mathrm{tr}{\tilde{c}}^{\left(i\right)}\right)\equiv \frac{\partial \Phi \left(\mathrm{tr}{\tilde{c}}^{\left(i\right)}\right)}{\partial \mathrm{tr}{\tilde{c}}^{\left(i\right)}}$$

meaning that the corresponding Volterra derivative of the free energy with respect to the conformation tensor is

(4b) $$\frac{\delta A}{\delta {\tilde{c}}^{\left(i\right)}}=\frac{G_{e}^{\left(i\right)}}{2}\left[h\left(\mathrm{tr}{\tilde{c}}^{\left(i\right)}\right)I-{\left({\tilde{c}}^{\left(i\right)}\right)}^{-1}\right]$$

Here, the following FENE-P(Wagner) expression is used:

(4c) $$h\left(\mathrm{tr}{\tilde{c}}^{\left(i\right)}\right)=\frac{b_{e}^{\left(i\right)}-3}{b_{e}^{\left(i\right)}-\mathrm{tr}{\tilde{c}}^{\left(i\right)}}$$

where $b_{e}^{\left(i\right)}$ is the finite extensibility (FENE) parameter of the entanglement strand associated with the i^th^ mode. As shown by the study of Stephanou et al. [35], $b_{e}=3\left[{\left(0.82\right)}^{2}/C_{\infty }\right]\left(M_{e}/M_{0}\right)$ (when all FENE parameters are considered equal), where $C_{\infty }$ is the polymer characteristic ratio at infinite chain length, *M_e_* is the entanglement molecular weight, and *M*_0_ is the average molar mass of a monomer. For example, for PS melts, *b_e_* = 54 [35].

### 2.3. The Poisson and Dissipation Brackets

Following the work of Beris and Edwards [36], the Poisson bracket for multiple modes is given as follows:

(5a) $$\begin{array}{ll} {\left\{F,G\right\}}_{c}= & -\sum_{i=1}^{N}\int \left[\frac{\delta F}{\delta c_{a\beta }^{\left(i\right)}}\nabla_{\gamma }\left(c_{a\beta }^{\left(i\right)}\frac{\delta G}{\delta M_{\gamma }}\right)-\frac{\delta G}{\delta c_{a\beta }^{\left(i\right)}}\nabla_{\gamma }\left(c_{a\beta }^{\left(i\right)}\frac{\delta F}{\delta M_{\gamma }}\right)\right]dV\\ & +\sum_{i=1}^{N}\int c_{\gamma a}^{\left(i\right)}\left[\frac{\delta F}{\delta c_{a\beta }^{\left(i\right)}}\nabla_{\gamma }\left(\frac{\delta G}{\delta M_{\beta }}\right)-\frac{\delta G}{\delta c_{a\beta }^{\left(i\right)}}\nabla_{\gamma }\left(\frac{\delta F}{\delta M_{\beta }}\right)\right]dV\\ & +\sum_{i=1}^{N}\int c_{\gamma \beta }^{\left(i\right)}\left[\frac{\delta F}{\delta c_{a\beta }^{\left(i\right)}}\nabla_{\gamma }\left(\frac{\delta G}{\delta M_{\alpha }}\right)-\frac{\delta G}{\delta c_{a\beta }^{\left(i\right)}}\nabla_{\gamma }\left(\frac{\delta F}{\delta M_{\alpha }}\right)\right]dV \end{array}$$

We note both here and throughout this paper that Einstein’s summation convention for repeated Greek indices is employed. The complete Poisson bracket was then simply given as follows:

(5b) $$\begin{array}{ll} \left\{F,G\right\}= & -\int \left[\frac{\delta F}{\delta M_{\gamma }}\nabla_{\beta }\left(M_{\gamma }\frac{\delta G}{\delta M_{\beta }}\right)-\frac{\delta G}{\delta M_{\gamma }}\nabla_{\beta }\left(M_{\gamma }\frac{\delta F}{\delta M_{\beta }}\right)\right]dV\\ & +{\left\{F,G\right\}}_{c} \end{array}$$

Next, the following expression for the dissipation bracket associated with the conformation tensors is used [36].

(6) $$\begin{array}{l} {\left[F,G\right]}_{\mathrm{nec}}=-\sum_{i=1}^{N}\int \frac{\delta F}{\delta c_{a\beta }^{\left(i\right)}}\Lambda_{\alpha \beta \gamma \varepsilon }^{\left(ii\right)}\frac{\delta G}{\delta c_{\gamma \varepsilon }^{\left(i\right)}}dV\\ +\sum_{i=1}^{N}\int L_{\alpha \beta \gamma \varepsilon }^{\left(i\right)}\left[\frac{\delta F}{\delta c_{\gamma \varepsilon }^{\left(i\right)}}\nabla_{\alpha }\left(\frac{\delta G}{\delta M_{\beta }}\right)-\frac{\delta G}{\delta c_{\gamma \varepsilon }^{\left(i\right)}}\nabla_{\alpha }\left(\frac{\delta F}{\delta M_{\beta }}\right)\right]dV \end{array}$$

The first integral on the right-hand side of Equation (6) accounts for the relaxation effects of each conformation tensor, which is proportional to a fourth–rank relaxation tensor, whereas the second integral allows for the non-affine deformation of each conformation tensor. We note that the subscript “nec”, meaning “no entropy production correction”, is added to the dissipation bracket to indicate that this bracket lacks terms that involve Volterra derivatives with respect to the entropy density, which can be omitted when we consider isothermal systems [36]. We further note that although, in general, the dissipation bracket allows explicit coupling between cross modes, as shown in Equations (8.2–25) of Beris and Edwards [36], in this study, they are omitted for simplicity. Then,

(7a) $${\tilde{\dot{c}}}_{\alpha \beta ,[1]}^{\left(i\right)}=-\Lambda_{\alpha \beta \gamma \varepsilon }^{\left(ii\right)}\frac{\delta A}{\delta c_{\gamma \varepsilon }^{\left(i\right)}}+L_{\alpha \beta \gamma \varepsilon }^{\left(i\right)}\nabla_{\gamma }u_{\varepsilon }$$

where we have defined the upper-convected time derivative:

(7b) $${\tilde{\dot{c}}}_{\alpha \beta ,[1]}^{\left(i\right)}\equiv \frac{\partial {\tilde{c}}_{\alpha \beta }^{\left(i\right)}}{\partial t}+u_{\gamma }\nabla_{\gamma }c_{\alpha \beta }^{\left(i\right)}-{\tilde{c}}_{\alpha \gamma }^{\left(i\right)}\nabla_{\gamma }u_{\beta }-{\tilde{c}}_{\gamma \beta }^{\left(i\right)}\nabla_{\gamma }u_{\alpha }$$

Finally, the extra (polymeric) stress tensor is given as follows:

(8) $$\sigma_{\alpha \beta }=\sum_{i=1}^{N}\left(2c_{a\gamma }^{\left(i\right)}\frac{\delta A}{\delta c_{\gamma \beta }^{\left(i\right)}}+L_{\alpha \beta \gamma \varepsilon }^{\left(i\right)}\frac{\delta A}{\delta c_{\gamma \varepsilon }^{\left(i\right)}}\right)$$

### 2.4. The Matrices L and Λ 

The relaxation matrix of each mode $\Lambda_{\alpha \beta \gamma \varepsilon }^{\left(ii\right)}$ is split into two contributions, following the work of Stephanou et al. [23], which have different relaxation times:

(9a) $$\begin{array}{l} \Lambda_{\alpha \beta \gamma \varepsilon }^{\mathrm{rept},(ii)}=\frac{f_{\mathrm{rept}}^{\left(i\right)}\left(tr{\tilde{c}}^{\left(i\right)}\right)}{2G_{e}^{\left(i\right)}\tau_{\mathrm{CR}}^{\left(i\right)}}\left({\tilde{c}}_{\alpha \gamma }^{\left(i\right)}{\tilde{\beta }}_{\beta \varepsilon }^{\left(i\right)}+{\tilde{c}}_{\alpha \varepsilon }^{\left(i\right)}{\tilde{\beta }}_{\beta \gamma }^{\left(i\right)}+{\tilde{c}}_{\beta \gamma }^{\left(i\right)}{\tilde{\beta }}_{\alpha \varepsilon }^{\left(i\right)}+{\tilde{c}}_{\beta \varepsilon }^{\left(i\right)}{\tilde{\beta }}_{\alpha \gamma }^{\left(i\right)}\right)\\ \Lambda_{\alpha \beta \gamma \varepsilon }^{\mathrm{Rouse},(ii)}=\frac{f_{\mathrm{Rouse}}^{\left(i\right)}\left(tr{\tilde{c}}^{\left(i\right)}\right)}{2G_{e}^{\left(i\right)}\tau_{R}^{\left(i\right)}\left(tr\tilde{c}\right)}\left({\tilde{c}}_{\alpha \gamma }^{\left(i\right)}{\tilde{\beta }}_{\beta \varepsilon }^{\left(i\right)}+{\tilde{c}}_{\alpha \varepsilon }^{\left(i\right)}{\tilde{\beta }}_{\beta \gamma }^{\left(i\right)}+{\tilde{c}}_{\beta \gamma }^{\left(i\right)}{\tilde{\beta }}_{\alpha \varepsilon }^{\left(i\right)}+{\tilde{c}}_{\beta \varepsilon }^{\left(i\right)}{\tilde{\beta }}_{\alpha \gamma }^{\left(i\right)}\right) \end{array}$$

Here, $\tau_{\mathrm{CR}}^{\left(i\right)}$ is the CR relaxation time of the i^th^ mode, which is considered to be half of the corresponding reptation time, $\tau_{\mathrm{CR}}^{\left(i\right)}=\frac{1}{2}\tau_{d}^{\left(i\right)}$ [39] [we note that this time coincides with the CCR relaxation time at equilibrium, as shown in Equation (10)], and $\tau_{R}^{\left(i\right)}\left(tr\tilde{c}\right)$ is the Rouse relaxation time of the i^th^ mode,

(9b) $$\tau_{R}^{\left(i\right)}\left(tr\tilde{c}\right)=\tau_{R,\mathrm{eq}}^{\left(i\right)}{\left(\frac{tr{\tilde{c}}^{\left(i\right)}}{3}\right)}^{k^{\left(i\right)}}$$

where $\tau_{R,\mathrm{eq}}^{\left(i\right)}$ is the equilibrium Rouse relaxation time of the i^th^ mode, which is given as $\tau_{d}^{\left(i\right)}=3Z\tau_{R,\mathrm{eq}}^{\left(i\right)}$ [19], and $k^{\left(i\right)}$ is the Extended White–Metzner (EWM) exponent [36] for the i^th^ mode. We note that for the Rouse time, a shear rate dependency through the use of the trace of the conformation tensor of each mode is considered. The functions $f_{\mathrm{rep}}^{\left(i\right)}\left(tr{\tilde{c}}^{\left(i\right)}\right)$ and $f_{\mathrm{Rouse}}^{\left(i\right)}\left(tr{\tilde{c}}^{\left(i\right)}\right)$ are scalar functions of the trace of the conformation tensor, as defined via the following equation [23]:

(9c) $$f_{\mathrm{Rouse}}^{\left(i\right)}\left(tr{\tilde{c}}^{\left(i\right)}\right)=1-f_{\mathrm{rep}}^{\left(i\right)}\left(tr{\tilde{c}}^{\left(i\right)}\right)=\beta_{ccr}^{\left(i\right)}\frac{h\left(tr{\tilde{c}}^{\left(i\right)}\right)tr{\tilde{c}}^{\left(i\right)}-3}{3+\beta_{ccr}^{\left(i\right)}\left[h\left(tr{\tilde{c}}^{\left(i\right)}\right)tr{\tilde{c}}^{\left(i\right)}-3\right]}$$

where $\beta_{ccr}^{\left(i\right)}$ is the CCR parameter of the i^th^ mode. For the (dimensionless) mobility tensor ${\tilde{\beta }}^{\left(i\right)}$ of the i^th^ mode, the Giesekus’ postulate ${\tilde{\beta }}^{\left(i\right)}=I+\alpha^{\left(i\right)}{\tilde{\sigma }}^{\left(i\right)}$ is used [37] with ${\tilde{\sigma }}^{\left(i\right)}=\sigma^{\left(i\right)}/G_{i}$, and $\alpha^{\left(i\right)}$ is the anisotropic mobility (Giesekus) parameter of the i^th^ mode. Then, with $\Lambda_{\alpha \beta \gamma \varepsilon }^{\left(ii\right)}=\Lambda_{\alpha \beta \gamma \varepsilon }^{\mathrm{rep},(ii)}+\Lambda_{\alpha \beta \gamma \varepsilon }^{\mathrm{Rouse},(ii)}$, we obtain

(9d) $$\Lambda_{\alpha \beta \gamma \varepsilon }^{\left(ii\right)}=\frac{1}{2G_{e}^{\left(i\right)}\tau_{\mathrm{CCR}}^{\left(i\right)}\left(tr{\tilde{c}}^{\left(i\right)}\right)}\left({\tilde{c}}_{\alpha \gamma }^{\left(i\right)}{\tilde{\beta }}_{\beta \varepsilon }^{\left(i\right)}+{\tilde{c}}_{\alpha \varepsilon }^{\left(i\right)}{\tilde{\beta }}_{\beta \gamma }^{\left(i\right)}+{\tilde{c}}_{\beta \gamma }^{\left(i\right)}{\tilde{\beta }}_{\alpha \varepsilon }^{\left(i\right)}+{\tilde{c}}_{\beta \varepsilon }^{\left(i\right)}{\tilde{\beta }}_{\alpha \gamma }^{\left(i\right)}\right)$$

where [23,39] the CCR relaxation time is obtained as follows:

(10) $$\frac{1}{\tau_{\mathrm{CCR}}^{\left(i\right)}\left(tr{\tilde{c}}^{\left(i\right)}\right)}=\frac{1}{\tau_{\mathrm{CR}}^{\left(i\right)}}+\left(\frac{1}{\tau_{R}^{\left(i\right)}\left(tr{\tilde{c}}^{\left(i\right)}\right)}-\frac{1}{\tau_{\mathrm{CR}}^{\left(i\right)}}\right)\beta_{ccr}^{\left(i\right)}\frac{h\left(tr{\tilde{c}}^{\left(i\right)}\right)tr{\tilde{c}}^{\left(i\right)}-3}{3+\beta_{ccr}^{\left(i\right)}\left[h\left(tr{\tilde{c}}^{\left(i\right)}\right)tr{\tilde{c}}^{\left(i\right)}-3\right]}$$

Finally, the expression of the $L_{}^{\left(i\right)}$ matrix is given via the following equation [36,37]:

(11) $$L_{\alpha \beta \gamma \varepsilon }^{\left(i\right)}=-\frac{\xi^{\left(i\right)}}{2}\left({\tilde{c}}_{\alpha \gamma }^{\left(i\right)}\delta_{\beta \varepsilon }+{\tilde{c}}_{\alpha \varepsilon }^{\left(i\right)}\delta_{\beta \gamma }+{\tilde{c}}_{\beta \gamma }^{\left(i\right)}\delta_{\alpha \varepsilon }+{\tilde{c}}_{\beta \varepsilon }^{\left(i\right)}\delta_{\alpha \gamma }\right)$$

Here, $\xi^{\left(i\right)}$ is the non-affine/slip parameter of the i^th^ mode. This parameter is important, as it allows for the prediction of a transient stress undershoot (following the overshoot) at high shear rates [23].

### 2.5. Thermodynamic Admissibility

Any thermodynamic system must obey the restriction of a non-negative rate of total entropy production. When the fluid studied is isothermal and incompressible, the entropy production results from the degradation of the mechanical energy, leading to $dH_{m}/dt=\left[H_{m},H_{m}\right]\leq 0$ [36]. For this aspect to be satisfied in our model, the following equation must hold:

(12) $$\sum_{i=1}^{N}\frac{\delta F}{\delta c_{a\beta }^{\left(i\right)}}\Lambda_{\alpha \beta \gamma \varepsilon }^{\left(i\iota \right)}\frac{\delta G}{\delta c_{\gamma \varepsilon }^{\left(i\right)}}=\sum_{i=1}^{N}\frac{G_{e}^{\left(i\right)}}{2\tau_{\mathrm{CCR}}^{\left(i\right)}\left(tr{\tilde{c}}^{\left(i\right)}\right)}\sum_{k=1}^{3}\frac{{\left(h\mu_{k}^{\left(i\right)}-1\right)}^{2}}{\mu_{k}^{\left(i\right)}}\left[1+\alpha^{\left(i\right)}\left(1-\xi^{\left(i\right)}\right)\left(h\mu_{k}^{\left(i\right)}-1\right)\right]\geq 0$$

where $\mu_{k}^{\left(i\right)},k=\{1,2,3\},i=\{1,..,N\}$ are the three eigenvalues of the conformation tensor of the i^th^ mode. Obviously, since the conditions $0\leq \alpha^{\left(i\right)}\left(1-\xi^{\left(i\right)}\right)<1,0\leq \xi^{\left(i\right)}<1,\forall i$ and $\beta_{ccr}^{\left(i\right)}\geq 0,\forall i$ [23] guarantee that each term of the summation is positive, the sum as a whole is also positive, meaning that the multimode version of the Stephanou et al. model [23] presented in this work is thermodynamically admissible.

### 2.6. Conformation Tensor Evolution Equation

The evolution equation used for each of the dimensionless conformation tensors is obtained by substituting Equations (4b), (9d), and (11) into Equation (7a)

(13) $$\begin{array}{l} {\tilde{\dot{c}}}_{\left[1\right]}^{\left(i\right)}=-\frac{1}{\tau_{\mathrm{CCR}}^{\left(i\right)}\left(\mathrm{tr}{\tilde{c}}^{\left(i\right)}\right)}\{\alpha^{\left(i\right)}\left(1-\xi^{\left(i\right)}\right)h^{2}\left(\mathrm{tr}{\tilde{c}}^{\left(i\right)}\right){\tilde{c}}^{\left(i\right)}\cdot {\tilde{c}}^{\left(i\right)}\\ \;+\left[1-2\alpha^{\left(i\right)}\left(1-\xi^{\left(i\right)}\right)\right]h\left(\mathrm{tr}{\tilde{c}}^{\left(i\right)}\right){\tilde{c}}^{\left(i\right)}-\left[1-\alpha^{\left(i\right)}\left(1-\xi^{\left(i\right)}\right)\right]I\},\forall i\in [1,N] \end{array}$$

The CCR relaxation time for the i^th^ mode is given in Equation (10), and the (dimensionless) effective spring constant is given in Equation (4c). Finally, the expression for the polymeric stress tensor is obtained by substituting Equations (4b) and (11) into Equation (8) as follows:

(14) $$\sigma =\sum_{i=1}^{N}G_{e}^{\left(i\right)}\left[h\left(\mathrm{tr}{\tilde{c}}^{\left(i\right)}\right){\tilde{c}}^{\left(i\right)}-I\right]$$

## 3. Asymptotic Behavior of the Model in Steady State Shear

In this section, we provide analytical expressions that describe the asymptotic behavior of the multimode version of the Stephanou et al. [23] model in the limit of weak flows for the following cases: inception of simple shear flow (SSF) described by the kinematics $u=\left(\dot{\gamma }y,0,0\right)$, in which $\dot{\gamma }$ is the (constant) shear rate; inception of uniaxial elongation flow (UEF) described by the kinematics $u=\left(\dot{\varepsilon }x,-\frac{1}{2}\dot{\varepsilon }y,-\frac{1}{2}\dot{\varepsilon }z\right)$, in which $\dot{\varepsilon }$ is the (constant) elongation rate; and small amplitude oscillatory shear (SAOS) described by the kinematics $u=\left(\dot{\gamma }\cos\left(\omega t\right)y,0,0\right)$, in which *ω* is the oscillation frequency. The material functions to be analyzed are as follows: (a) the transient shear viscosity $\eta^{+}(t)$ (= $\sigma_{\mathrm{yx}}\left(t\right)/\dot{\gamma }$) and the first, $\Psi_{1}^{+}(t)$ (=$\left(\sigma_{\mathrm{xx}}(t)-\sigma_{\mathrm{yy}}(t)\right)/{\dot{\gamma }}^{2}$), and second, $\Psi_{2}^{+}(t)$ (=$\left(\sigma_{\mathrm{yy}}(t)-\sigma_{\mathrm{zz}}(t)\right)/{\dot{\gamma }}^{2}$), normal stress coefficients in the case of shear; (b) the transient elongational viscosity, $\eta_{E}^{+}(t)$ (=$\left(\sigma_{\mathrm{xx}}(t)-\sigma_{\mathrm{yy}}(t)\right)/\dot{\varepsilon }$), in the case of uniaxial elongation; and (c) the storage, $G^{\prime }\left(\omega \right)$, and loss, $G^{\prime \prime }\left(\omega \right)$, moduli in the case of SAOS. 

To obtain the asymptotic behavior, we need to expand the conformation tensor for each mode in the limit of small strain rates (by invoking a linearization of the evolution equation for each of the conformation tensors) and analytically solve the corresponding ordinary differential equations. After this stage, we obtain the non-zero stress tensor components via Equation (14). Finally, we obtain the following results for the relevant material functions:

*Inception of shear:*

(15a) $$\eta^{+}\left(t\right)=\sum_{i=1}^{N}\tau_{\mathrm{CR}}^{\left(i\right)}{\tilde{G}}_{e}^{\left(i\right)}\left[1-\exp\left(-\frac{t}{\tau_{\mathrm{CR}}^{\left(i\right)}}\right)\right]$$

(15b) $$\Psi_{1}^{+}\left(t\right)=2\sum_{i=1}^{N}{\left(\tau_{\mathrm{CR}}^{\left(i\right)}\right)}^{2}{\tilde{G}}_{e}^{\left(i\right)}\left[1-\left(1+\frac{t}{\tau_{\mathrm{CR}}^{\left(i\right)}}\right)\exp\left(-\frac{t}{\tau_{\mathrm{CR}}^{\left(i\right)}}\right)\right]$$

(15c) $$\begin{array}{r} -\Psi_{2}^{+}\left(t\right)=\sum_{i=1}^{N}{\left(\tau_{\mathrm{CR}}^{\left(i\right)}\right)}^{2}{\tilde{G}}_{e}^{\left(i\right)}\{\left[\xi^{\left(i\right)}+\alpha^{\left(i\right)}\left(1-\xi^{\left(i\right)}\right)\right]\left[1-\left(1+\frac{t}{\tau_{\mathrm{CR}}^{\left(i\right)}}\right)\exp\left(-\frac{t}{\tau_{\mathrm{CR}}^{\left(i\right)}}\right)\right]\;\\ \alpha^{\left(i\right)}{\left(1-\xi^{\left(i\right)}\right)}^{2}\exp\left(-\frac{t}{\tau_{\mathrm{CR}}^{\left(i\right)}}\right)\left[1-\frac{t}{\tau_{\mathrm{CR}}^{\left(i\right)}}-\exp\left(-\frac{t}{\tau_{\mathrm{CR}}^{\left(i\right)}}\right)\right]\} \end{array}$$

*Inception of uniaxial elongation:*

(16) $$\eta_{E}^{+}\left(t\right)=3\sum_{i=1}^{N}\tau_{\mathrm{CR}}^{\left(i\right)}{\tilde{G}}_{e}^{\left(i\right)}\left[1-\exp\left(-\frac{t}{\tau_{\mathrm{CR}}^{\left(i\right)}}\right)\right]$$

meaning that Trouton’s law is true for the steady-state extensional viscosity. 

*Small Amplitude Oscillatory Shear*:

(17) $$\begin{array}{l} G^{\prime }\left(\omega \right)=\sum_{i=1}^{N}{\tilde{G}}_{e}^{\left(i\right)}\frac{{\left(\omega \tau_{\mathrm{CR}}^{\left(i\right)}\right)}^{2}}{1+{\left(\omega \tau_{\mathrm{CR}}^{\left(i\right)}\right)}^{2}}\\ G^{\prime \prime }\left(\omega \right)=\sum_{i=1}^{N}{\tilde{G}}_{e}^{\left(i\right)}\frac{\omega \tau_{\mathrm{CR}}^{\left(i\right)}}{1+{\left(\omega \tau_{\mathrm{CR}}^{\left(i\right)}\right)}^{2}} \end{array}$$

In Equations (15)–(17), we have defined ${\tilde{G}}_{e}^{\left(i\right)}\equiv {\left(1-\xi^{\left(i\right)}\right)}^{2}G_{e}^{\left(i\right)}$. 

## 4. Results and Discussion

**Figure 1:**
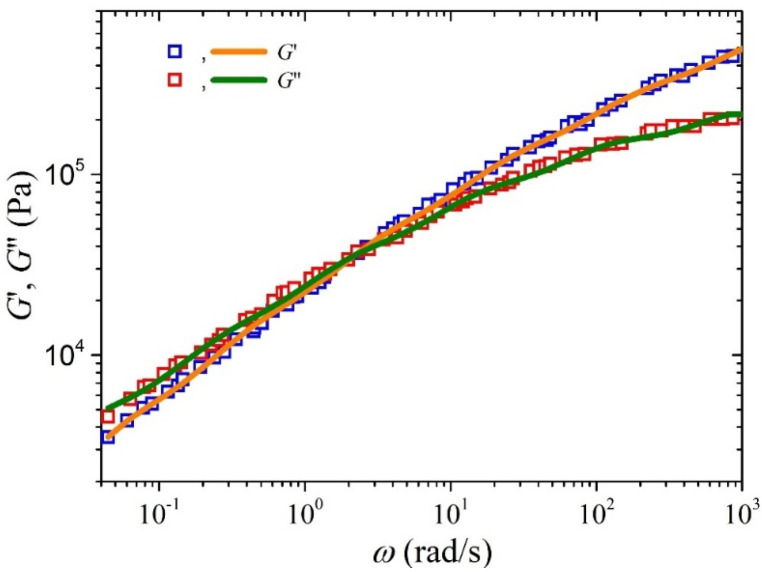
Comparison between the model predictions Equation (17) and the experimental data presented in Ref. [15] for the storage and loss moduli of an HDPE sample at 200 °C. The relaxation spectrum is provided in Table 1.

The FENE parameter can be easily calculated via $b_{e}=3{\left(0.82\right)}^{2}M_{e}/\left(M_{0}C_{\infty }\right)$, as mentioned above. In this study, PE *M*_0_ = 14 g/mol, whereas *C*_∞_ = 7.3 and *M_e_* = 828 g/mol (see Table 3.3, p. 151 of Ref. [40]). These values yield *b_e_* = 16.34. We will compare against the experimental data of Konaganti et al. [15] that have performed rheological measurements of the sample HDPE-1 (reported by the same group [41]), for which *M_w_* = 206 kg/mol; thus, the number of entanglements is equal to *Z* ≈ 249 >> 1. The relaxation spectrum is the same as the one used by Konaganti et al. [15] (see their paper’s Table 2 for *T* = 200 °C, though is also provided in Table 1), and it was obtained by fitting the expressions of the storage and loss moduli, which are shown in Equation (17), with the corresponding experimental data. The comparison against the experimental storage and loss moduli is shown in Figure 1.

**Figure 2:**
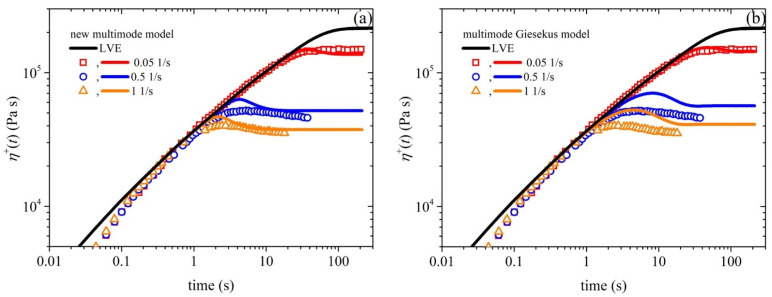
(**a**) Model prediction of the growth of the shear viscosity prediction (lines) upon the inception of the shear flow at three different dimensionless shear rates, along with comparison with the experimental data (symbols) considered in Ref. [15]. In panel (**b**), we depict the same comparison for the multimode Giesekus model. The thick black line depicts the LVE envelope, which is shown in Equation (15a).

All of the remaining parameter values are obtained by fitting the model predictions with the experimental data; for simplicity, we assume that each parameter has the same value for all modes, although, in general, different values for each mode could be considered (e.g., Konaganti et al. [15]). The following values of the model parameter are chosen to provide a good comparison with the start-up shear flow experimental data provided in Figure 2: *ξ* = 0.02, *α* = 0.3, *β*_ccr_ = 4 × 10^−4^, and *k* = −3.5. For comparison, we also depict, in the following figures, the predictions of the multimode Giesekus model [which is a special case of our model in which *β*_ccr_ = *ξ* = *k* = 0 and *b_e_* infinite or the function *h* = 1 in Equation (4c)] with *α* = 0.3 and the relaxation spectrum of Table 1.

### 4.1. Comparison with Start-Up Shear Flow Data

**Figure 3:**
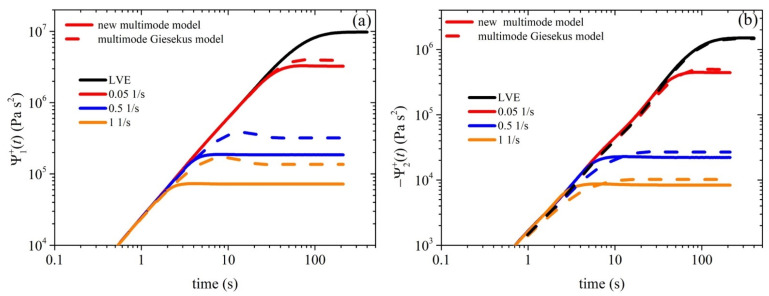
Model predictions (red, blue, and orange lines) of the growth of the first (**a**) and second (**b**) normal stress coefficients upon the inception of shear flow. The thick black lines depict the LVE envelope, which is shown in Equations (15b) and (15c). The s parameter values are the same as those used in in Figure 2. The multimode Giesekus model’s predictions are also depicted (red, blue, and orange dashed lines). The thick black dashed lines depict the LVE envelope, which are again shown in Equations (15b) and (15c).

**Figure 4:**
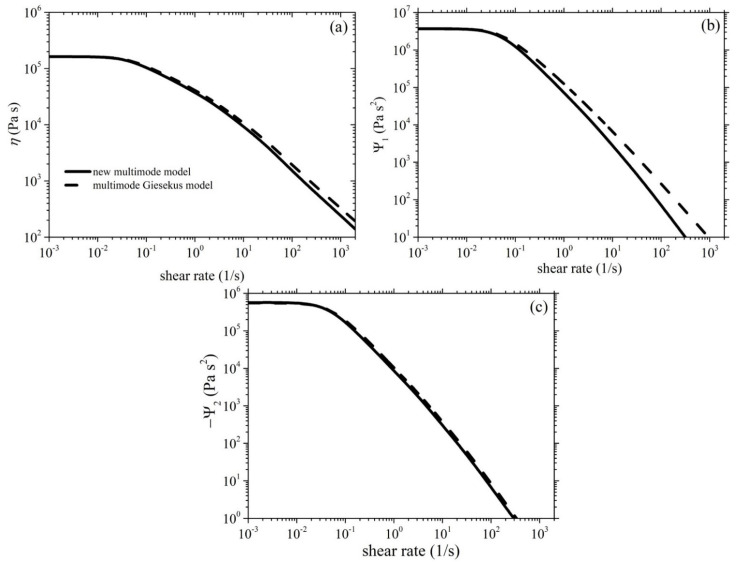
Model predictions of the steady-state (**a**) shear viscosity and the (**b**) first and (**c**) second normal stress coefficients of the HDPE-1 sample. The parameter values are the same as those used in Figure 2. The multimode Giesekus model prediction is also depicted (black dashed lines).

Figure 2a illustrates the comparison between the experimental data for the growth of the shear viscosity upon inception of shear flow and the simulated results obtained using the new model. The experimental data (symbols) were collected at three different shear rates—0.05 1/s, 0.5 1/s, and 1 1/s—whereas the lines represent the simulated shear viscosity values at the corresponding shear rates. It can be observed that the model accurately captures the trends and magnitude of the shear viscosity over time, doing so much more successfully than the Giesekus model (Figure 2b). We should, however, note that the overshoots noted at the two larger shear rates (0.5 1/s and 1 1/s) are higher than the experimental data. The overshoot predicted using our model is controlled by two parameters: the FENE parameter *b_e_* and the anisotropic mobility (Giesekus) parameter *α*. As mentioned above, the former parameter is not a free parameter, as it is directly dictated by structural parameters. The latter parameter is a free parameter, and by increasing its value, the start-up shear viscosity overshoots noted at the two larger shear rates (0.5 1/s and 1 1/s) shift downwards and broaden (results not shown), thus more closely agreeing with the experimental data; however, the good comparison identified at the smaller shear rate (0.05 1/s) is reduced. This result might hint that the parameter α should not be a constant, but should increase with the applied strain rate. Similar arguments have been put forth and resulted in a variable non-affine/slip parameter proposed by Nikiforidis et al. [42] and a variable link tension coefficient proposed by Stephanou and Kröger [26]. We note that although a non-zero value of *ξ* is employed, the undershoots produced are too small to be noted via the scale used in Figure 2. Although no experimental data are provided, we provide the corresponding prediction of the growth of the first and second normal stress coefficients in Figure 3, as well as the steady-state values of all viscometric material functions in Figure 4.

### 4.2. Comparison with Start-Up Uniaxial Elongational Flow Data

**Figure 5:**
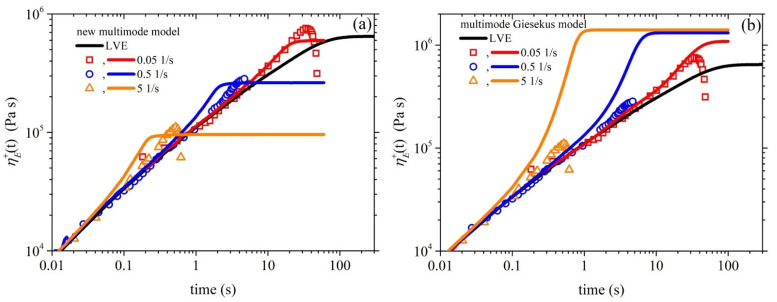
(**a**) Comparison between the model predictions (lines) and the experimental measurements (symbols) of Konaganti et al. [15] for the growth of the elongational viscosity as a function of time for several stretch rates. The thick black line depicts the LVE envelope, as given in Equation (16). The sparameter values are the same as those used in Figure 2 except *ξ* = 0. In panel (**b**), we depict the same comparison for the multimode Giesekus model.

**Figure 6:**
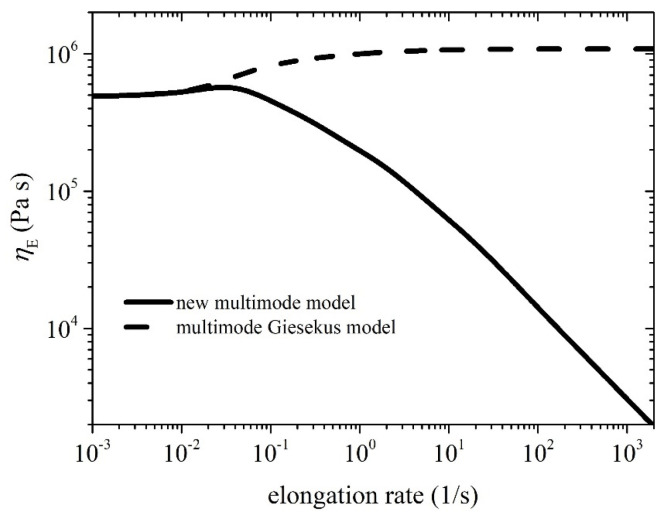
Model prediction of the steady-state uniaxial elongational viscosity. The parameter values are the same as those used in Figure 2 except *ξ* = 0. The multimode Giesekus model prediction is also depicted (black dashed line).

In Figure 5, we provide the comparison between the model predictions and the experimental measurements obtained by Konaganti et al. [15] for the growth of the elongational viscosity as a function of time upon inception of uniaxial elongation at the following stretch rates: 0.05 1/s, 0.5 1/s, and 5 1/s. The comparison employs the same parameter values as those used in Figure 2, except *ξ* = 0, as informed by Stephanou et al. [37], and the corresponding steady-state prediction is provided in Figure 6. Given that the parameter values were selected based on the start-up shear data (Figure 2), the comparison is noted to be adequately appropriate. We note that the Giesekus model (Figure 5b) fails to reproduce correctly the trend of the experimental data. It should be noted that the uniaxial elongation data obtained by Konaganti et al. [15] do not seem to reach a steady state. This outcome is customary in the literature [11,12,43] due to experimental difficulties, as the polymer samples used become too slender and often break. This issue has led some researchers to consider the highest value as the steady-state elongational viscosity and omit all following data [44]. Thus, the data that follow the overshoot may not be accurate. Overall, the proposed model demonstrates good agreement with the experimental data, indicating its effectiveness in describing the rheological behavior of polymers of industrial significance. 

## 5. Conclusions

In this work, we developed the multiple-mode version of a generalized, conformation-tensor based viscoelastic model for polymer melts, which was proposed in [23], by making use of the generalized bracket formalism of Beris and Edwards [36]. Like its forerunner, i.e., the single-mode version [23], it accounts for the most significant effects that can be realized in entangled polymer systems: anisotropic drag, finite extensibility, non-affine motion (leading to the exhibition of a transient stress undershoot at large shear rates), and variable chain relaxation due to convective constraint release. The multiple-mode version of the model has been shown to have a very good predictive capability with regard to the industrial experimental data for HDPE obtained by Konaganti et al. [15]. Obviously, a better comparison could have been obtained if we had simultaneously fitted all available data, as Konaganti et al. [15] did (we note that some parameters must have different values in different flows, such as the non-affine/slip parameter, which must be explicitly considered in the fitting process). Furthermore, different values of the model parameters for each mode could also have been considered, following the work of Konaganti et al. [15], which would certainly provide more flexibility to the model and, thus, improve its capacity to fit the experimental data. However, in our present work, we mostly focused on deriving the multimode version of the model proposed by Stephanou et al. [23] using non-equilibrium thermodynamics, with less focus devoted to its predictive capacity to almost perfectly fit all available experimental data.

The model, in its present form, only considers strictly linear chains. Industrial samples, particularly LDPE, are never strictly linear, having either short or long branches distributed along their entire backbone. There is clear evidence that all material functions of PE are considerably affected by the presence of even low levels of long-chain branching [45]. We, therefore, need to generalize it and allow the explicit consideration of branches, following the guidelines provided by the pom-pom [27] model and its thermodynamically admissible version, known as the pom-pon [28] model. Furthermore, the multimode version does not explicitly consider the molecular weight (MW) distribution of industrial samples, such as the log-normal or gamma distributions, that are able to describe experimentally measured distributions [46]. As such, we should also generalize it to handle molecular weight distribution, following the work of Schieber [47,48]. This generalized constitutive model will allow a more accurate prediction of the rheological responses of industrially used polymeric systems that possess an extensive spectrum of MW. Finally, we used an ambiguous model-fitting process wherein the values of the model parameters were obtained to best compare them to the experimental data. However, atomistic non-equilibrium molecular dynamics simulations can be used to directly obtain some of the parameters from the simulations [37,49]. For example, the EWM exponent can be obtained by directly comparing the prediction of Equation (9b) to the relaxation time as a function of shear rate at large shear rates obtained via the NEMD simulations, and the CCR parameter can be obtained by directly comparing the average number of entanglements as a function of shear rate which, due to CCR, is noted to decrease [22] (we note, however, that in our present work we omitted this approach and considered a constant number of entanglements). Only then, polymer engineers would be able to accurately use the predictions of the multimode constitutive model for comparison with the rheological response noted in actual industrial processes. Our findings provide a foundation for future research that aims to enhance the properties of high MW polymers for diverse applications.

## Figures and Tables

**Table 1:** Relaxation spectrum [15].

Mode	${\tilde{G}}_{e}^{\left(i\right)}\;(\mathrm{Pa})$	$\tau_{\mathrm{CR}}^{\left(i\right)}\;(s)$
1	387,808	0.00086
2	185,307	0.0075
3	93,338	0.0548
4	37,766	0.403
5	12,934	2.99
6	5025	30.78

## Data Availability

Not applicable.
